# Glucose metabolism tests and recurrent pregnancy loss: evidence from a systematic review and meta-analysis

**DOI:** 10.1186/s13098-022-00973-z

**Published:** 2023-01-05

**Authors:** Sedigheh Hantoushzadeh, Omid Kohandel Gargari, Arman Shafiee, Niloofar Seighali, Marjan Ghaemi

**Affiliations:** 1 Vali-E-Asr Reproductive Health Research Center, Imam Complex, Family Health Research Institute, Tehran University of Medical Sciences, East Bagherkhan Ave, Tehran, Iran; 2 Student Research Committee, School of Medicine, Alborz University of Medical Sciences, Karaj, Iran

**Keywords:** Recurrent pregnancy loss, Diabetes, Systematic review, Glucose metabolism

## Abstract

**Objective:**

To synthesize the published citations to determine the association between glucose metabolism tests and recurrent pregnancy loss (RPL).

**Method:**

The electronic databases including PubMed, Scopus and Web of Science were searched for the original articles that evaluated the correlation between glucose metabolism tests including fasting blood glucose (FBG), fasting insulin (FI), homeostatic model assessment for insulin resistance (HOMA-IR), the rate of individuals with HOMA-IR  > 4.5, insulin resistance, fasting glucose/fasting insulin (FG/FI) and FG/FI  > 4.5.and recurrent pregnancy loss with a combination of proper keywords.

**Results:**

The database search led to finding 390 articles. Detailed screening of titles and abstracts for potential eligibility was performed, and after excluding the duplicated and irrelevant citations, finally, 8 studies were selected to be included in this study, 7 observational studies and one controlled clinical trial. A significant difference in the amount of FI, HOMA-IR, the rate of HOMA-IR  > 4.5, the rate of individuals with insulin resistance, fasting glucose/fasting insulin (FG/FI), and the rate of FG/FI  > 4.5 were found among RPL patients compared to controls. There was no difference when comparing FBG between the groups.

**Conclusion:**

This study indicates an important link between abnormal glucose metabolism tests and a history of recurrent pregnancy loss. These data may encourage clinicians to request glucose metabolism tests other than FBG in women with recurrent pregnancy loss.

**Supplementary Information:**

The online version contains supplementary material available at 10.1186/s13098-022-00973-z.

## Introduction

Recurrent pregnancy loss (RPL) is defined as the failure of two or more consecutive pregnancies before 20–24 weeks of gestation [1] and is experienced by 2.5% of women trying to conceive [2]. However, it must be noted that the classical definition of RPL includes 3 or more losses [3, 4]. The causes may include immunologic and endocrinology disorders, genetic factors, infections, uterine malformation, as well as low quality of the embryo or gametes [5, 6]. Although, no risk factors are identified in more than half of the women [7]. It is believed that diabetes or glucose intolerance as an endocrine abnormality could be a possible risk factor for RPL [6, 8]. Women with RPL have high fasting insulin and insulin resistance [9].

One of the most critical questions that remain to be answered is if or how glucose metabolism affects pregnancy loss. Hence, we decided to carry out a systematic review and meta-analysis to improve our understanding of the relationship between glucose metabolism tests, including fasting blood glucose (FBG), fasting insulin (FI), homeostatic model assessment for insulin resistance (HOMA-IR), the rate of individuals with HOMA-IR  > 4.5, insulin resistance, fasting glucose/fasting insulin (FG/FI) and FG/FI  > 4.5 and RPL. This knowledge might contribute to understand the factors associated to RPL that can be the focus of prevention and management strategies.

## Materials and methods

This study was conducted in accordance with the guidance presented in the Cochrane.

Handbook for Systematic Reviews of Interventions and reported according to the Preferred Reporting Items for Systematic Reviews and Meta-Analysis (PRISMA) statement (Additional file 1: Table S1) [10]. This study did not require ethical approval. Our protocol has been prospectively conducted and submitted with PROSPERO, The International Prospective Register of Systematic Reviews.

### Study setting

This systematic review was conducted to integrate and improve our understanding of the relationship between glucose metabolism tests including fasting blood glucose (FBG), fasting insulin (FI), homeostatic model assessment for insulin resistance (HOMA-IR), the rate of individuals with HOMA-IR  > 4.5, insulin resistance, fasting glucose/fasting insulin (FG/FI) and FG/FI  > 4.5 and RPL.

### Inclusion and exclusion criteria

Inclusion criteria based on PICOT acronyms were: (1) population: adult women; (2) exposure: patients with a history of two or more pregnancy losses defined as RPL; (3) control: patients with a history of at least one live birth as the control group; (4) outcome: evaluation of different glucose metabolism tests; (5) study design: all original articles which including observational cohorts and case–control studies. Only English articles were assessed. Exclusion criteria were as follows: pregnancy loss due to extrinsic causes, review articles, opinion pieces or guidelines, non-peer-reviewed papers, unpublished reports, and articles in which the date and location of the study were not specified.

### Search strategy

The initial search was undertaken in 3 main databases including PubMed, Scopus and Web of Science until 1 May 2022. For the search strategy, combinations of the following keywords (Habitual Abortion OR Recurrent Abortion OR Recurrent Miscarriage OR Recurrent Early Pregnancy Loss) AND (Diabetes OR Glucose intolerance OR Impaired Glucose Tolerance OR Hyperglycemia OR Insulin Resistance OR Glucose Metabolism Disorders OR Insulin Sensitivity OR Hyperinsulinemia OR Metabolic Syndrome OR HOMA-IR OR Insulin Insufficiency or Fasting Blood Sugar OR Fasting Blood Glucose) as medical subject heading (Mesh) terms were used.

### Study registration

The protocol of this study was registered and approved by the ethics review board of Tehran University of Medical Sciences.

### Study selection

Titles and abstracts were independently retrieved and reviewed for eligibility by two authors (MG and AS) and non-relevant studies and studies that did not meet the inclusion criteria were excluded. After the initial screening, the remaining full texts were reviewed by SH; articles with not sufficient data and duplicates were identified. Eligibility of included studies and cases reported in case series were double checked at this stage.

### Quality assessment, risk of bias

NS and AS independently assessed the quality of included studies using the National Institutes of Health (NHLBI/NIH) quality assessment tools for case–control, cross-sectional studies, and randomized controlled trials [11]. Studies scoring 9 or more were marked as “Good”, studies scoring between 7 and 8 were marked as “Fair” and studies rating less than 7 were marked as “Poor”.

### Data extraction

NS extracted data from included studies to excel.

### Outcomes

We investigated 7 potential markers that were used to assess glucose metabolism test in the included studies: (1) fasting blood glucose (FBG); (2) fasting insulin (FI); (3) homeostatic model assessment for insulin resistance (HOMA-IR); (4) the rate of individuals with HOMA-IR > 4.5; (5) the rate of individuals with insulin resistance; (6) fasting glucose/fasting insulin (FG/FI); (7) the rate of individuals with FG/FI  > 4.5. The main outcome was to find the association between glucose metabolism tests and RPL.

### Data analysis

For outcomes with I^2^ > 25% random effect model, and for outcomes with I^2^ < 25% fixed effect model was used for data pooling. For continuous outcomes, Standard mean difference (SMD) was used for reporting combined results. SMD is more generalizable than regular mean difference even when using similar units [12]. For dichotomous outcomes, odds ratio was the selected measure for summarization. Leave-on-out sensitivity analysis was done to detect an effect of individual studies on the overall results. Data analysis was done with RevMan 5.4. Data presented in graphs were extracted using Plot Digitizer software.

## Results

### General outcomes

**Fig. 1:**
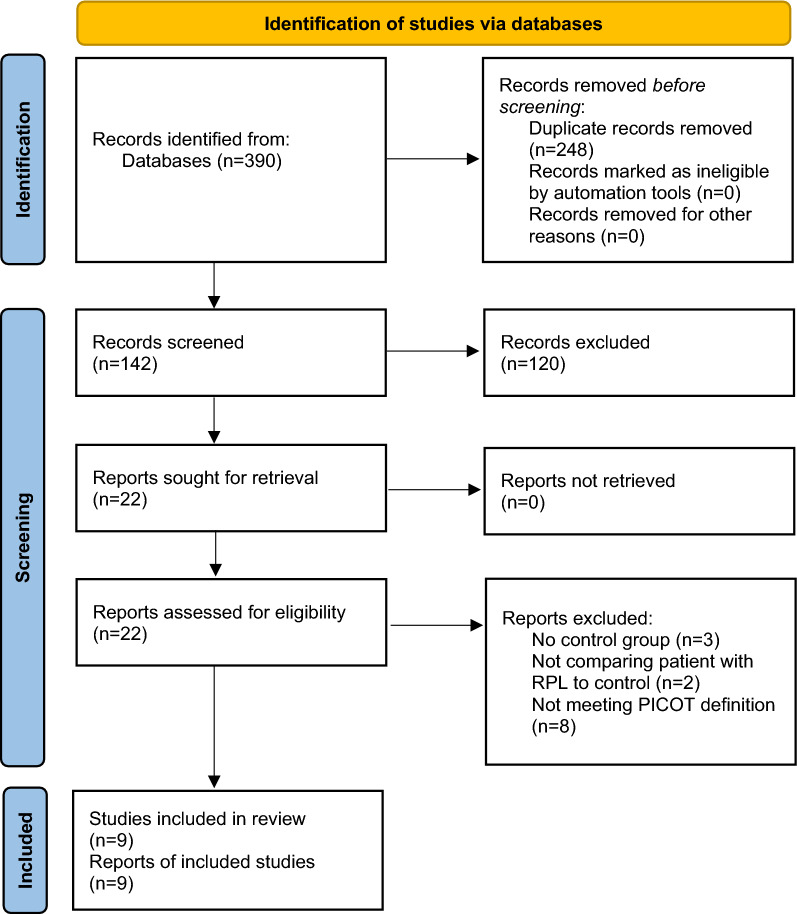
PRISMA flow chart of the study selection and screening process

The database search led to finding 390 articles. Detailed screening of titles and abstracts for potential eligibility was performed, 248 were duplicated, and the irrelevant papers were removed. According to inclusion and exclusion criteria, the full texts of 22 articles were reviewed. Three studies did not have a control group and were excluded. Two other prospective cohort studies considered diabetes and insulin resistance as exposures and RPL as an outcome, so we excluded these studies because they were not comparing patients with a history of RPL to patients without a history of RPL [13, 14]. Finally, 9 studies [8, 9, 15–21] were selected to be included in this study. Figure 1 presents the PRISMA flow chart of the study selection and screening process.

Seven studies reported combinable outcomes and were used for quantitative synthesis and meta-analysis. Study by Habets et al. [17] reported some combinable outcomes but they chose median and interquartile range rather than mean and standard deviation as their measure of central tendency so we could not pool their findings with other studies, and only report them separately. Study by Zolghadri et al. [19] was the only study reporting glucose tolerance test (GTT) as an outcome and did not report any combinable outcome so this study was not included in meta-analysis.

### Characteristics of included studies

All studies excluded patients with a history of diabetes or metabolic syndrome [8, 9, 15, 16, 18, 20–22] except for one study by Habets et al. [17]. Two studies included pregnant women only [16, 18] but other studies included non-pregnant women [8, 9, 15, 17, 19–22]. Table 1 summarizes the characteristics of the included studies.

**Table 1:** Characteristics of included studies

1st author	Year	Country	Study type	Participants (n)	Time interval	Mean age	Combinable outcomes	RPL definition	Quality
Craig [8]	2002	USA	Single center, case-controlled, prospective study	RPL (74), Control (74)	April 2000–January 2001	Case group: 32.7 ± 5.4/Control group: 32.8 ± 6.0/P = 0.92	FBS, FI, FG/FI, HOMA-IR > 4.5	2 or more	Good
Zolghadri [19]	2007	Iran	Prospective control clinical trial	RPL (164), Control (74)	August 2003–June 2005		Not combinable	3 or more	Fair
Kotanaie [20]	2012	Iran	Prospective case–control study	RPL (50), Control (50)	March 2007–March 2008	Case group: 28.02 ± 4.57/Control group: 28.28 ± 4.77/p = 0/5715	FBS, FI, FG/FI	3 or more	Fair
Diejomaoh [15]	2007	Kuwait	Cross-sectional study	RPL (35), Control (30)	October 2002–June 2004	study group: 28.6 ± 4.3/Control group: 27.5 ± 4.6	FBS, FI, HOMA-IR, HOMA-IR > 4.5, FG/FI	3 or more	Fair
Wang [18]	2011	China	Cross-sectional study	RPL (97), Control (52)	June 2008–September 2010	patient group: 30.81 ± 4.01/control group: 29.15 ± 4.62	FBS, FI, HOMA-IR > 4.5,	2 or more	Fair
Edugbe [16]	2020	Nigeria	Multicenter cross-sectional study	RPL (80), Control (80)	NR	Case: 28.09 ± 6.14/Control: 28.10 ± 6.21	FBS, FI, HOMA-IR, HOMA-IR > 4.5,	2 ore more	Good
Habets [17]	2022	Netherlands	Retrospective case–control study	Case group: 123 women with either a history of low-order RPL (2–3 pregnancy losses) (65 patients) or high-order RPL (≥ 4 pregnancy losses) (58 patients)/ Control group: 20 healthy women with a history of at least one previous uncomplicated pregnancy	2015–2019	Group of RPL = 2 + 3: 32.8[29.5–35.4]/Group of RPL ≥ 4: 33.7[30.4–36.5]/control: 32[29.5–38]/p = 0.390	FBS, FI	2 or more	Good
Ispasoiu [9]	2013	Romania	Single center, case–control study	65 patients in case group and 53 patients in control	2011–2012	Case: 30.12 (4.904) Control: 29.36 (5.274)	FBS, FI, HOMA-IR	2 or more	Fair
Wani [21]	2017	Pakistan	Single center, case-controlled	Case: 75 Non pregnant with history of 2 or more recurrent pregnancy losses Control: 75 non pregnant women with no RPL with at least one live birth	2014–2015	Case: 28.4 (2.37) Control: 29.1(2.7)	FBS, FI, HOMA-IR, HOMA-IR > 4.5, FG/FI, FG/FI < 4.5	2 or more	Fair

*RPL* recurrent pregnancy loss

### Risk of bias

Two studies were labeled as “Good” and seven others were labeled as “Fair”. Detailed results of quality assessment for each study have been summarized in Additional file 1: Table S1.

### Fasting blood glucose

**Fig. 2:**
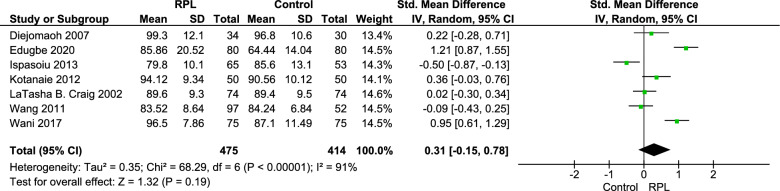
Forest plot of pooled standard mean fasting blood glucose (FBG) difference

Seven studies, including 889 patients (475 cases and 414 controls), reported fasting blood glucose (FBG) as their outcome. FBG was slightly higher in patients with a history of RPL compared to the control group, but this difference was not significant. Pooled standard mean difference (SMD) was 0.31 (CI 95% = [− 0.15–0.78], P = 0.19) with high heterogeneity (I^2^ = 91%, Tau2 = 0.35) (Fig. 2).

A subgroup analysis based on the inclusion of pregnant women was done. In both subgroups, FBG was higher in RPL group (not significant). Pooled estimated for subgroup of studies that included pregnant women and subgroup of studies that did not include pregnant women are the following respectively: SMD = 0.56, CI 95% = [− 0.71–1.84], P = 0.39 and SMD = 0.21 CI 95% = [− 0.28–0.71], P = 0.40. Inclusion or exclusion of pregnant women does not seem to have a significant effect (Test for between-subgroup differences P = 0.62) (Additional file 1: Fig. S1).

Another subgroup analysis based on definition of RPL was done. Interestingly in RPL ≥ 3 subgroup FBG was significantly higher among RPL patients (SMD = 0.31, CI 95% = [0.00–0.61]. However, this difference between the subgroups does not seem to have a significant (P = 0.97) (Additional file 1: Fig. S2).

Habets et al. [17] study result was consistent with our findings, this study compared fasting blood glucose between three groups, patients who had 2 or three pregnancy losses, patients who had more than 4 pregnancy losses and control group, the results were the following: group of 2–3 RPL: 5.0 [4.75–5.4] mmol/L/Group of RPL ≥ 4: 4.95 [4.8–5.3] mmol/L/control: 4.9 [4.75–5.15] mmol/ (p = 0.7).

### Fasting insulin

**Fig. 3:**
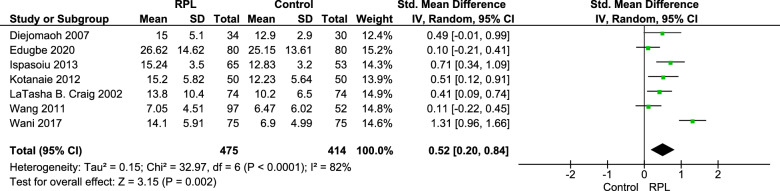
Forest plot of pooled standard mean difference of fasting insulin

Fasting insulin (FI) level was reported in 7 studies including 889 participants (475 cases and 414 controls) and the results showed a significant increase in insulin level of patients with history of RPL (Pooled SMD = 0.52 [0.20,0.84],${I}^{2}=82\%, Tau2=0.15, p=0.002$) (Fig. 3).

Habets et al. [17] reported fasting insulin in median and interquartile range and result was the following, Group of 2–3 RPL: 39.7 [26.9–57.6] pmol/L, group of RPL ≥ 4: 36.4 [24.3–65.7] pmol/L, control: 46.5 [29.5–49.3] pmol/L, p = 0.939.

### Fasting blood glucose (FG)/Fasting insulin (FI)

**Fig. 4:**
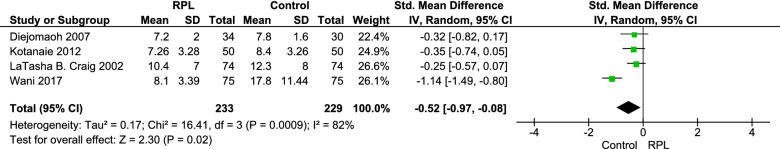
Forest plot of pooled standard mean difference fasting blood glucose (FG)/fasting insulin (FI)

This outcome was reported in 4 studies including 462 patients (233 cases and 229 controls). This outcome was significantly lower among RPL patients compared to control SMD = − 0.52 [− 0.97, − 0.08], ${I}^{2}=82\%, Tau2=0.17, p=0.02$ (Fig. 4).

In three studies, number of patients with FG/FI < 4.5 were reported. Combining these two studies, we calculated the pooled odds ratio, the result is significant, OR = 3.26, CI 95% = [1.58, 6.74], P = 0.001 (Additional file 1: Fig. S3).

### Homeostatic model assessment for insulin resistance (HOMA-IR)

**Fig. 5:**
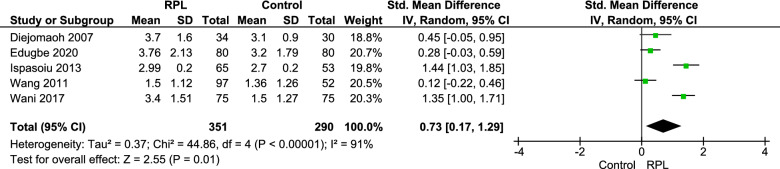
Forest plot of pooled standard mean HOMA-IR difference

HOMA-IR was reported in 5 studies, including 641 patients (351 cases, 290 controls). HOMA-IR was significantly higher among patients with history of RPL, SMD = 0.73, CI 95% = [0.17–1.29], p = 0.01, I^2^ = 91% (Fig. 5).

Number of patients with HOMA-IR more than 4.5 was reported in three studies including 362 patients (183 cases and 179 controls). Poold OR was 3.51, P = 0.0001, I^2^ = 0% (Additional file 1: Fig. S4).

### Glucose tolerance test (GTT)

Zolghadri et al. [19] in a randomized clinical trial compared glucose tollerance test (GTT) between non pregnant patients with history of RPL(n = 133) and control(n = 70) group. Patients with blood glucose levels between 140 and 200 mg/dl after ingestion of 75 g glucose were classified as impaired GTT. 17.6% of patients with RPL history had impaired GTT, 5.4% of control group had impaired GTT, OR = 1.34, CI 95% = 0.25–2.42, P = 0.17.

### Sensitivity analysis

We performed a sensitivity analysis by excluding the results of each study to assess how their findings have effect on the overall results. Table 2 presents the results of leave-one-out sensitivity analysis.

**Table 2:** leave-one-out sensitivity analysis for outcomes with more than 3 studies. Pooled estimates of each outcome after leaving out each study on the left column are presented

Left-out study	Pooled estimates			
	FBS	FI	HOMA-IR	FG/FI
Diejomaoh [4]	SMD = 0.33 [-0.20, 0.85], I^2^ = 93%, P = 0.22	SMD = 0.52 [0.16, 0.89], I^2^ = 85%, P = 0.005	SMD = 0.79 [0.12, 1.46], I2 = 93%, P = 0.02	SMD = − 0.58 [− 1.15, − 0.01], I2 = 87%, P = 0.05
Edugbe [16]	SMD = 0.16 [-0.25, 0.57], I^2^ = 87%, P = 0.44	SMD = 0.59 [0.25, 0.94], I^2^ = 80%, P = 0.0008	SMD = 0.84 [0.16, 1.53], I2 = 92%, P = 0.02	NR
Kotanaie [20]	SMD = 0.30 [-0.24, 0.84], I^2^ = 93%, P = 0.27	SMD = 0.52 [0.14, 0.90], I^2^ = 85%, P = 0.007	NR	SMD = − 0.58 [− 1.19, 0.02], I2 = 87%, P = 0.06
Craig [8]	SMD = 0.36 [− 0.18, 0.90], I^2^ = 92%, P = 0.19	SMD = 0.54 [0.15, 0.93], I^2^ = 85%, P = 0.006	NR	SMD = − 0.62 [− 1.19, − 0.05], I2 = 83%, P = 0.03
Wang [18]	SMD = 0.38 [− 0.14, 0.91], I^2^ = 92%, P = 0.16	SMD = 0.59 [0.24, 0.94], I^2^ = 82%, P = 0.0010	SMD = 0.88 [0.27, 1.50], I2 = 90%, P = 0.005	NR
Ispasoiu [9]	SMD 0.45 [0.00, 0.90], I^2^ = 89%, P = 0.05	SMD = 0.49 [0.12, 0.86], I^2^ = 84%, P = 0.01	SMD = 0.55 [− 0.02, 1.12], I2 = 90%, P = 0.06	NR
Wani [21]	SMD = 0.20 [− 0.28, 0.69], I^2^ = 91%, P = 0.41	SMD = 0.37 [0.17, 0.57], I^2^ = 44%, P = 0.0003	SMD = 0.57 [− 0.00, 1.14], I2 = 89%, P = 0.05	SMD = − 0.30 [0 −0.52, 0− 0.07], I2 = 0%, P = 0.009

## Discussion

The present study, to our knowledge, was the first systematic review and meta-analysis to assess the relationship between recurrent pregnancy loss (RPL) and glucose intolerance. We found a significant difference in the amount of FI, HOMA-IR, the rate of HOMA-IR  > 4.5, the rate of individuals with insulin resistance, FG/FI, and the rate of FG/FI  > 4.5 among RPL patients compared to controls. There was no difference when comparing FBG between the groups.

Several factors have been linked to the etiology of RPL. However, the incidence of unexplained RPL is about 40% in most studies [9, 15, 23]. PCOS is the most reported endocrine disorder in women, and it is claimed that insulin resistance and hyperinsulinemia are one of the main causes of the pathophysiology of RPL [24].

In the 9 studies included in this systematic review, 7 studies with 889 individuals reported the FBG in both patients with RPL and controls. We found a non-significant association between FBG and recurrent miscarriages. The majority of the reports on this topic are in line with our findings [8, 15, 17, 18]. Recently, a study by Habets et al. aimed to evaluate vascular and metabolic status of women with RPL and women with a history of uncomplicated pregnancy [17]. They found that there was no significant difference between FBG and RPL. In contrast to these findings, Edugbe et al. found that women with RPL had a higher mean FBG [16].

Two studies by Crane et al. [14] and McCoormack [13] et al. were excluded from our study but their results are helpful. Crane et al. compared pregnancy loss between diabetic and non-diabetic patients; they reported no significant difference between these two groups. And McCormack et al. reported a high rate of hyperinsulinemia among patients with recurrent pregnancy loss.

Our findings suggest that there is a significant association between recurrent miscarriages and IR in patients with RPL by calculating the pooled SMD of HOMA-IR as a potential biomarker for assessing IR in patients. This finding was further confirmed by analyzing the odds of HOMA-IR  > 4.5 between the RPL and comparing group.

Consistent with our results, Ispasoiu [9] and Tian et al. [25] found that in patients with RPL, the mean level of HOMA-IR was significantly higher compared to normal subjects, although both of these studies evaluated a small sample size. These contradictory findings were explained by Wang et al. [18]. Different approaches were used in their investigation to examine how the IR status between the case and control groups differed. Between the 5th and 13th weeks of pregnancy, they performed an oral glucose tolerance test (OGTT) and an insulin-releasing test. They found that only a short time after consuming the oral glucose solution did the two groups significantly differ from one another (Based on 1-, 2-, and 3-h of both glucose and insulin tests). Furthermore, they stated that no significant differences between the levels of FBG, FI, and HOMA-IR were observed when comparing two groups, indicating that relying solely on these indicators to detect the presence of IR in RPL-affected women is inadequate.

The findings of our meta-analysis suggested that patients with RPL have significantly higher mean fasting insulin (FI) levels than controls. These results suggest that hyperinsulinemia may be present in women who experience recurrent miscarriages. Hyperinsulinemia is associated with decreased expression levels of glycodelin and insulin-like growth factor binding protein 1 (IGFBP-1), which are responsible for the immune response of the mother’s immune system towards the implanted embryo and facilitation of the adhesion process of blastocysts, respectively [9, 26, 27]. It is also suggested that hyperinsulinemia could increase the level of plasminogen activator inhibitor-1 and induce villous thrombosis then causing trophoblastic hypoplasia and miscarriage.

Although the gold standard for identifying IR is the clamp technique [28], this test is not widely used by clinicians because of its time-consuming procedure and complex nature [15]. Therefore, most of the studies used the fasting glucose to fasting insulin (FG/FI) ratio and the HOMA-IR. In our study, we identified a significant increase in both the mean of FG/FI and the odds of FG/FI > 4.5 among women with RPL.

### Clinical implication

Although there is some debate about the exact biomarker alterations that occur in women who have had a history of RPL, the majority of studies agree that these women experience increased IR. The screening of pregnant mothers with a history of RPL in terms of metabolic status and blood sugar level should be prioritized to investigate possible IR states. FI, HOMA-IR, and FG/FI as IR indicators in these individuals need to be assessed in light of our findings.

### Strengths and limitations

Our study has several strengths. We performed a comprehensive systematic review of three databases in line with the PRISMA statement with no date and language restrictions. To adjust serum levels of different markers for assessing IR, pooled effect sizes of relevant outcomes were calculated using the inverse variance method.

Despite these strengths, we also faced number of limitations. The most important one lies in the fact that most of the included studies were observational, leading to the fact that these studies are limited-designed in nature and carry inherent biases. The majority of the studies included in the meta-analysis were of fair quality, which could result in weakening the strength of this meta-analysis. The paucity of studies in this field may contribute to publication bias.

In conclusion, our study revealed a significant association between abnormal glucose metabolism tests and a history of recurrent pregnancy loss. These findings may persuade medical professionals to order further glucose metabolism tests in women who experience repeated miscarriages in addition to FBG.

## Supplementary Information

**Additional file 1. Table S1.** PRISMA 2020 checklist. **Table S2.** Quality assessment for each included study. **Figure S1.** Subgroup analysis based on the inclusion of pregnant women in the study population. **Figure S2.** Subgroup analysis based on the definition of recurrent pregnancy loss (RPL). **Figure S3.** Forest plot for patients with Fasting blood glucose (FG) / Fasting insulin (FI) <4.5. **Figure S4.** Forest plot for patients with Homeostatic Model Assessment for Insulin Resistance (HOMA-IR) >4.5.

## Data Availability

The datasets used during the current study all are included in the manuscript.
